# Youth cannabis use in Canada post-legalization: service providers’ perceptions, practices, and recommendations

**DOI:** 10.1186/s13011-023-00550-1

**Published:** 2023-06-22

**Authors:** Toula Kourgiantakis, Eunjung Lee, A. Kumsal Tekirdag Kosar, Christine Tait, Carrie K.Y. Lau, Sandra McNeil, Shelley Craig, Rachelle Ashcroft, Charmaine C. Williams, Abby L. Goldstein, Uppala Chandrasekera, Deepy Sur, J. L. Henderson

**Affiliations:** 1 Factor-Inwentash Faculty of Social Work, University of Toronto, 246 Bloor Street West, Toronto, ON M5S 1V4 Canada; 2 Department of Applied Psychology and Human Development, Ontario Institute for Studies in Education, Toronto, ON Canada; 3 Ontario Association of Social Workers (OASW), Toronto, ON Canada; 4 Margaret and Wallace McCain Centre for Child, Youth and Family Mental Health, Department of Psychiatry, Centre for Addiction and Mental Health (CAMH), University of Toronto, Toronto, ON Canada

**Keywords:** Youth, Cannabis, Legalization, Canada, Service providers, Mixed methods

## Abstract

**Background:**

In 2018, Canada legalized recreational cannabis use with the purpose of protecting youth and restricting access. However, concerns have been raised that this objective has not been met as rates of cannabis use among youth aged 16–24 have not declined. Youth cannabis use is associated with various adverse effects including psychosis, anxiety, depression, suicidality, respiratory distress, cannabinoid hyperemesis syndrome, and intoxications. Service providers play a crucial role in addressing youth cannabis use. This study aimed to understand Ontario service providers’ perceptions, practices, and recommendations on youth cannabis use.

**Methods:**

This mixed method study included a survey and two focus groups. The survey was distributed to mental health service providers serving youth aged 16–24 across Ontario who were given the option to participate in a focus group. The survey included closed and open-ended questions regarding perceptions, practices, and recommendations, while the focus groups explored these categories in greater depth. Descriptive statistics were used to analyze close-ended questions and interpretative content analysis was applied for open-ended questions. Focus group data were analyzed using thematic analysis.

**Results:**

The survey was completed by 160 service providers and 12 participated in two focus groups. Regarding perceptions, 60% of survey participants agreed with legalization, 26% had a strong understanding of medical versus recreational cannabis, 84% believed that cannabis has physical and mental health risks, and 49% perceived stigmatization. Less than half of the survey participants reported screening or assessing cannabis use, 16% stated they are highly familiar with treating cannabis use, and 67% reported that they rarely work with families. Subthemes identified in the focus groups under perceptions included normalization and stigmatization, harms for youth, and stigma, racism, and discrimination. Subthemes under practice included cannabis not being the primary focus, challenges with screening, assessment, and intervention, and referral to specialized services. Both the survey and focus group participants recommended increasing public education, enhancing service provider training, improving regulation and policies, reducing stigma and minimization, improving service access, and providing more culturally responsive services.

**Conclusion:**

Youth cannabis use in Canada remains a significant public health concern, necessitating a more comprehensive plan to protect Ontario youth and reduce associated harms.

Canada has one of the highest prevalence rates of cannabis use in the world [1], and cannabis use is highest among young adults aged 20–24 (50%) followed by 16–19-year-olds (37%) [2]. In 2018, Canada legalized recreational cannabis use with the implementation of the Cannabis Act – a national policy intended to guide the selling and distribution of cannabis across the country [3]. The purpose of the Cannabis Act is to protect public health and safety, with a specific focus on a few key areas, including to “protect the health of young persons by restricting their access to cannabis” [3]. However, researchers report that to date, the objective of protecting youth has not been met since the Cannabis Act has not led to a reduction in youth cannabis use, and youth cannabis use remains a serious public health concern [4].

Since legalization came into effect, the rates of cannabis use in Canadian youth have increased [4–9] and there are increased rates of cannabis use disorder diagnoses in 18–24 -year-olds since legalization [10]. The cannabis market has expanded considerably since legalization, although there is large variation in cannabis access across the provinces. Ontario uses a private retail model and has 1,552 cannabis stores, compared to the neighbouring province of Quebec which uses a public model and has 91 cannabis stores [11]. Youth report easier access to cannabis [8, 12], with 41% of Ontario students in grades 7–12 reporting through the Ontario Student Drug and Health Survey (OSDUHS) that it is easy to obtain cannabis [13].

A study examining patterns of cannabis use among Canadian youth found that there is a high propensity for youth using cannabis at baseline to switch to multimode use (smoking, eating/drinking, vaping) or increase their use if already engaged in multimode use [5]. Some studies also report increased rates of initiation of cannabis use post-legalization among youth who did not use cannabis pre-legalization [8, 9]. There are also reports of increased use of cannabis among youth during the COVID-19 pandemic [2, 6, 14, 15], attributed to stress, anxiety, boredom, loneliness, and lack of a regular schedule [2]. One study found increased rates of cannabis use among 14–18-year-olds during the pandemic [6] and another reported that emerging adults who were self isolating used 20% more cannabis [14].

Several studies have reported that one of the most frequently reported motives for cannabis use by youth is coping with a range of concerns including feeling depressed and anxious [14, 16, 17]. Cannabis use among youth is associated with several adverse effects including poor wellbeing and psychosocial functioning [18, 19], psychosis [10, 20–24], anxiety [19, 25], depression [14, 18, 19, 26], and an increased risk of suicidality [27]. There are also high rates of polysubstance use [28] with binge drinking, vaping, and cannabis use as the most prevalent combination [29]. Cannabis use in youth has also been linked to physical health effects, including impaired cognitive performance, respiratory distress, lung injury, myocardial ischemia, seizures, oral health issues, weight loss, and cannabinoid hyperemesis syndrome [30]. Additionally, since the legalization of cannabis, there has been an increase in cannabis intoxications in young children and youth, along with cannabis-related emergency department visits [31–34]. Myran and colleagues (2022) found that increased emergency visits by youth related to cannabis use were linked with greater access to cannabis retail stores and increased commercialization [35].

Early cannabis initiation and use are associated with increased mental health and substance use challenges in adulthood. Hawke et al. [36] found that 30% of youth seeking clinical services started using cannabis before the age of 14, and these youth had increased rates of trauma, internalizing and externalizing symptoms, polysubstance use, and precarious housing. The adverse effects of youth cannabis use are highlighted in a recent comprehensive review that provides lower-risk cannabis guidelines and recommends delaying the initiation of cannabis use until after late adolescence to reduce adverse health effects [37].

There has been ample substance use research showing the relationship between an individual’s knowledge or perceptions of risks and how this influences their substance use [38–41]. One of the other purposes of the Cannabis Act is to “enhance public awareness of the health risks associated with cannabis use.” However, there are also shortcomings when examining the statistics on public awareness. The Canadian Cannabis Survey (CCS), developed by Health Canada, examines detailed information about patterns of cannabis use, as well as knowledge, attitudes and opinions related to cannabis use. It is conducted annually, and the 2022 survey found that 48% of Canadians have not noticed any education campaigns or public health messages on cannabis, and 52% have not noticed health warning messages on cannabis products. Furthermore, only 11% of Canadians have noticed public health messages about cannabis in health care settings [2]. In November 2022, the Canadian Centre on Substance Use and Addiction (CCSA) participated in a public consultation on cannabis legalization and underlined that there is misinformation and low cannabis literacy among Canadians. They noted that when there is limited understanding of risks and harms “people in Canada are not empowered to make informed choices about their cannabis health”.[42 p6-7] The report recommends more public education to reach priority populations such as youth because current public education efforts have been unsuccessful in reaching targeted populations.

There is a call for more education about cannabis use in youth, cannabis use prevention, early intervention, and treatment initiatives [36, 42], as well as training of service providers who play an important role screening, assessing, intervening, and providing psychoeducation on cannabis use to youth and their families. The Canadian Pediatric Society underlines the critical role of healthcare providers in regularly screening for recreational cannabis use with youth and responding to questions and concerns by parents nonjudgmentally using validated tools, as well as Motivational Interviewing and harm reduction approaches for youth to consider and reflect on their own cannabis use [43]. A survey conducted in the United States found variation and gaps in primary care providers’ knowledge and beliefs regarding cannabis, and many service providers described discomfort in discussing cannabis use with patients [44]. Muzyk et al. [45] conducted a scoping review on interprofessional substance use disorder education in health profession education programs and 14 studies met eligibility criteria. The review found that substance use disorder education improves students’ knowledge, beliefs, and attitudes toward substance use disorders (SUDs) which can influence patient outcomes. The authors noted that there were gaps in the literature, with most studies examining general substance use or tobacco. They recommended that future studies focus on students’ behaviours in clinical practice and the influence of interns’ behaviours on patient outcomes. They also recommended increased training on harm reduction strategies, as well as education on specific substances, especially those with greater public health concerns.

There has been limited research on Canadian service providers’ perceptions and practices related to youth cannabis use. There is also limited information on how professional training programs (e.g., social work or psychology programs) are preparing trainees for practice. There have been a few studies evaluating training and education programs, but they are either not youth-specific (e.g. see [46]) or they focus on cannabis for medical purposes (e.g. see [47]). Considering the high rates of cannabis use in Canadian youth and the potential harms and risks, it is important to increase our understanding of service providers’ perceptions and practices related to youth cannabis use. To address these gaps, the following research questions guided this study: (1) What are service providers’ perceptions of cannabis use in youth? (2) How do service providers describe their practice and approaches related to youth cannabis use? (3) What do service providers recommend at the policy, practice, and/or education levels to address cannabis use more effectively?

## Method

### Design and setting

This study was conducted using a mixed methods convergent design to achieve a more complete understanding of the research problem through anonymous survey data and two focus groups. We followed a six-step framework developed by Creswell & Hirose [48] for conducting a mixed methods study using survey research, which included the following steps: (1) explaining the rationale for mixed methods, (2) detailing quantitative and qualitative databases, (3) identifying a mixed method design, (4) analyzing and presenting the results of the quantitative and qualitative data, (5) presenting and showing integration, and (6) articulating the benefits of using mixed methods. For this study, quantitative data were gathered through an online, anonymous survey sent to mental health service providers across Ontario. Qualitative data were collected through open-ended questions on the survey and concurrently through virtual focus groups. The study was conducted in Ontario, which is the most populous province in Canada with over 15 million residents [49]. Ethics approval was obtained by the Research Ethics Board at the University of Toronto.

### Participant sample and recruitment

A purposeful sampling strategy that included both convenience and snowball sampling was used for this study. This type of sampling allows researchers to obtain information from participants who are knowledgeable in the phenomenon of interest, share similar characteristics, and are easily accessible [50, 51]. Eligible participants included service providers serving youth aged 16–24 in the broad field of mental health and addictions, such as social workers, psychologists, physicians, nurses, guidance counsellors, social service workers, child and youth workers, addiction counsellors, and psychotherapists. The 16–24 age group was selected because it represents an important developmental stage of transition between childhood and adulthood, and it is during this stage that most mental illnesses and substance use concerns emerge [52]. Some services in Ontario are offering services specifically for this age group, referred to as “transition-aged youth” or “emerging adults” [53, 54]. For consistency and simplicity, we used the term “youth” in our materials and interviews. Participants were recruited using a flyer that was distributed through social media sites, professional associations, and mental health agencies. The flyer included a link to the information and consent form, and after participants provided consent, they were given access to the anonymous survey. Survey participants were given the option to participate in the focus group, and those who completed the survey could provide their name and contact information to enter a draw for a $50 gift card. Their contact information was separated from their survey responses to preserve anonymity. Participants in the focus groups received a $30 gift card as an honorarium for their time.

### Data collection

Data collection for the online survey occurred from January to May 2022. We used Qualtrics, an online survey tool, to build the survey and collect the data [55]. The online survey questions and the focus group interview guide were developed in partnership with the research team, consisting of various experts in the field of mental health and addictions. The demographic section of the online survey consisted of 27 questions that asked about participant characteristics, geographic location, professional qualifications, and practice experience. The remainder of the survey focused on youth cannabis use, with 39 closed and open-ended questions grouped into three categories: 1) knowledge, beliefs, and attitudes about cannabis use in youth, 2) screening, assessment, and intervention, and 3) recommendations to strengthen policies, practice, education, and training.

Pilot testing is recommended to ensure the survey directions and questions are understood by participants, and to also elicit feedback on the appropriateness of wording [56]. We piloted the survey with members of the research team and mental health professionals who were part of our target population. Two 90-minute focus groups were conducted on a secure online platform on March 24 and 25, 2022. We developed a semi-structured interview guide with open-ended questions in the same three categories as the survey. Focus group participants completed a demographic questionnaire prior to the focus groups, and all interviews were audio recorded with participants’ consent. The interviews were transcribed and de-identified by assigning a participant ID code to preserve anonymity.

### Data analysis

The survey data were exported from Qualtrics to Excel, and three members of the research team analyzed the survey data (CT, AKTK, and TK). For closed responses, we analyzed the data using descriptive statistics, and for open-ended questions, we used interpretative content analysis [57, 58]. The quantitative survey data were analyzed in Excel using pivot tables, and the qualitative survey data were analyzed in Dedoose by two independent coders (CT, AKTK) who inductively generated coded data to interpret the meaning of the responses. The lead author (TK) reviewed the codes and met with the research assistants (CT, AKTK) to discuss codes, resolve discrepancies, and identify overarching themes. The focus group transcripts were organized, synthesized, and analyzed in Dedoose by two research assistants (RAs) and the principal investigator (PI: SM, CKYL, and TK) using thematic analysis [59, 60]. Thematic analysis is a six-stage process that includes: 1) familiarization of data; 2) development of initial codes; 3) identification of initial themes from coded data; 4) review of themes; 5) definition and development of names for themes; and 6) interpretation and reporting [59].

In the first phase of the focus group analysis, two RAs (SM & CKYL) and the PI (TK) familiarized themselves with the data by reading the transcripts and writing memos. We created a codebook that provided detailed descriptions of the codes with exemplars. In the second phase, the RAs identified initial codes that emerged from their review. This was a recursive process that involved constant movement between generating and defining codes, reading through transcripts, and adding and refining codes as needed [59]. The transcripts were coded by a first coder and reviewed by a second coder. The PI reviewed each double-coded transcript to resolve discrepancies and ensure consensus. We debriefed at our weekly meetings, discussed discrepancies, and updated our codebook. When coding was completed, we reviewed the codes with their excerpts and identified overarching themes and emergent subthemes.

## Results

### Survey results

A total of 160 service providers completed the survey (Table 1). Participants represented eight professions, with the highest proportion being social workers (69%), followed by Registered Psychotherapists (12%), social service workers (6%), child and youth workers (4%), Registered Nurses (3%), psychologists (1%), guidance counsellor (1%), physician (1%), and unspecified (5%). Regarding years of experience, 55% of service providers reported 10 years or less experience working in the field of mental health and addictions, while 45% had more than 11 years of experience. Most participants identified as women (75%), 12% identified as men, 9% as gender diverse including nonbinary, trans, gender fluid or gender queer, 3% preferred not to answer, and 1% selected that none of the identities listed represented them.

Participants spanned various age groups, with 23% under 29, 34% between 30–39, 19% between 40–49, 17% between 50–59, and 7% were  60 years of age or older. Geographically, participants were distributed across all five regions of Ontario, with 43% in Central Ontario (the most populated region of the province), 21% from Southwestern Ontario, 14% from Eastern Ontario, 10% from Northeastern Ontario, 7% from Northwestern Ontario, and 4% who identified living in more than one region. In terms of racial identity, more than two-thirds identified as white (69%), followed by Black (8%), mixed or biracial (8%), South Asian (3%), Indigenous (3%), Middle Eastern (2%), East/Southeast Asian (1%), Latinx (1%), another race category (3%), and 4% did not respond. Two-thirds of participants (66%) had their primary employment setting in the public sector, 28% were in a private setting, and 6% were in both public and private settings. The participants’ work settings included community agency (29%), private practice (24%), hospital (15%), primary care clinic (7%), residential treatment centre (2%), university (1%), high school (1%), more than one setting (16%), and other settings (5%).

**Table 1:** Survey participant characteristics (*N* = *160*)

Characteristics	Survey participants n (%)
Profession	
Social worker	110 (69%)
Registered psychotherapist	19 (12%)
Social service worker	9 (6%)
Child and youth worker	6 (4%)
Registered nurse (RN, RPN, RNA)	4 (3%)
Psychologist	2 (1%)
Guidance Counsellor	1 (1%)
Physician	1 (1%)
Other	8 (5%)
Experience working in health, mental health and/or addictions	
5 years or less	42 (26%)
6–10 years	47 (29%)
11–20 years	44 (28%)
21 + years	27 (17%)
Gender	
Woman	120 (75%)
Man	19 (12%)
Gender diverse (queer, gender fluid/queer, male queer, female queer, non-binary, gender independent, gender diverse, trans man/trans masculine/man of trans experience)	14 (9%)
Prefer not to answer	5 (3%)
None of these identities represent me	2 (1%)
Age	
20–29 years old	37 (23%)
30–39 years old	55 (34%)
40–49 years old	30 (19%)
50–59 years old	27 (17%)
60–69 years old	10 (6%)
70–79 years old	1 (1%)
Racial Identity	
Black (African, Afro-Caribbean, African Canadian)	12 (8%)
Mixed race	12 (8%)
South Asian	5 (3%)
Indigenous (First Nations, Métis, Inuit)	4 (3%)
Middle Eastern	3 (2%)
East/Southeast Asian	2 (1%)
Latino/Latina/Latinx	1 (1%)
White	110 (69%)
Prefer not to answer	6 (4%)
Another race category	5 (3%)
Religion	
No religion	68 (43%)
Christian	50 (31%)
Jewish	9 (6%)
Muslim	8 (5%)
Buddhist	1 (1%)
Indigenous Spirituality	1 (1%)
Hindu	1 (1%)
Another religion or spiritual affiliation	5 (3%)
Multiple religions selected	5 (3%)
Prefer not to answer	12 (8%)
Region	
Central Ontario	69 (43%)
Southwestern Ontario	34 (21%)
Eastern Ontario	23 (14%)
Northeastern Ontario	16 (10%)
Northwestern Ontario	11 (7%)
Multiple regions selected	7 (4%)

*Note*. Percentages may not total 100 due to rounding. Survey participant characteristics were self-reported.

All participants completed the survey, which consisted of 39 questions under categories related to perceptions, practices, and recommendations on youth cannabis use. In this section, we present some of the most salient responses to the quantitative survey under three categories: perceptions, practices, and recommendations. 

#### Service providers’ perceptions

More than half of the participants (60%) agree or strongly agree with cannabis legalization, 20% disagree or strongly disagree, while 20% of participants neither agreed nor disagreed about legalization. Less than half of the participants (43%) disagree or strongly disagree with the idea that cannabis is a gateway drug for use of other drugs and alcohol for youth, more than one-quarter (26%) agree or strongly agree, and 28% were in the middle. Only 26% of participants reported having a high level of familiarity with the differences and similarities between medical and recreational cannabis use, 49% reported a moderate understanding, 24% reported a low understanding, and 1% stated that they do not know. In terms of understanding the link between cannabis use and mental health, 48% of participants reported a high level of understanding, 49% reported a moderate level of understanding, and 3% reported a low level of understanding.

Most participants (84%) believe that cannabis use poses risks to youth physical and mental health, and almost two-thirds (65%) of participants believe that cannabis is harmful for youth. Regarding the question on benefits of cannabis use, 36% of participants do not believe that cannabis has benefits for youth, 27% believe that there are benefits, and 34% neither agree nor disagree about benefits. More than two-thirds of participants (68%) believe that cannabis use in youth is a serious public health concern and 36% believe that youth cannot use cannabis regularly without developing a cannabis use problem, 34% believe that youth can use cannabis regularly without developing a problem, and 27% neither agree nor disagree with this statement.

More than two-thirds of participants (72%) believe that there are inadequate services for youth using cannabis, 68% do not believe that addiction services are adequately integrated with mental health services, and 28% believe that cannabis use in youth should be treated primarily by an addiction specialist. Most participants (87%) reported having a high to moderate level of familiarity with the impact of social determinants of health for youth using cannabis, and 79% believe that services for youth using cannabis need to be culturally adapted. Most participants (89%) believe that families are an importance source of support, and 76% believe families should be involved in services and treatment for youth using cannabis. In response to an open-ended question on service providers’ perceptions of how stigma affects youth using cannabis, almost half of the participants (46%) perceive that youth using cannabis are stigmatized, 38% do not think youth cannabis use is stigmatized, 11% were unsure, and 5% did not respond. Table 2 provides some of the salient themes related to service providers’ perceptions.

**Table 2:** Service providers’ perceptions related to youth cannabis use from survey data

Topics	Frequency	Exemplar Quotes
Legalization of cannabis	60% agree 20% neither agree nor disagree 20% disagree	“I will support the legalization, when there is proper education in place.” “Since legalization, the rates in youth seem to be increasing, and since the pandemic the mental health of youth has really declined. Legalization appears to be associated with safe and harm free. We need to pull back on the promotion of cannabis.” “I agree with decriminalization but strongly disagree with the way this was legalized. It was for financial reasons and did not have a clear public health plan to prevent harms to children and youth. They did the same thing with vaping. It took years to reduce cigarette smoking rates, and it is irresponsible of our government and public health units.”
Understanding of cannabis used for medical purposes versus recreational use	49% moderate level of understanding 26% high level of understanding 25% low level of understanding	“Cannabis is not medical, and the government and pharmaceutical companies have convinced us that it is a form of medical treatment.” “Cannabis is widely considered normal and a rite of passage for youth. It is also legal (for adults) and even considered a medical treatment, natural, ‘good for you’ by many people in Canada. As such, youth tend to think it’s not a big deal to use it often and/or to self-medicate.”
Risks and adverse effects of cannabis use on youth	65% believe cannabis is harmful 84% believe there are risks to physical & mental health	“I have observations about how it affects [youth]. But I would like more scientific evidence. Many clients claim it treats their mental health issues, I feel it exacerbates mental health issues in the long term and makes it challenging to do therapeutic work if they are actively using.” “A more theoretical understanding of the risks of cannabis use by youth and young adults. Education and treatment regarding Cannabis Use Disorder and other mental health issues associated with cannabis. The majority of my young male clients have significant cognitive and social issues associated with their habitual and excessive cannabis use.” “I think the service providers, youth, parents, and the public do not have enough information on the risks associated with cannabis use. When I try to educate my patients, I am going against a narrative that is very strong about cannabis being beneficial/medical/natural.”
Importance of family support & involvement in treatment	89% agree about the importance of family support 76% believe families should be involved	“Substance use can be a symptom of a broader issue, and family is a key source of belonging and support. Especially if the youth/young adult is living with family, they need to be on the same page with the youth/young adult in their process so they can know how to be that source of support.” “Our addiction and mental health services do not support families and physicians cannot provide treatment for the whole family.”
Stigma of cannabis use	46% perceive cannabis use as stigmatizing 38% perceive there is no stigma 11% were unsure 5% did not respond	“A lot of people look down upon cannabis use still and there are a lot of stereotypes associated with cannabis users.” “The labelling by family that they are “lazy”, “irresponsible” which affects self worth and feeling hopeless.” “I think there is less of a stigma since legalization.” “No stigma because cannabis use is normalized. You are considered odd if you consider cannabis to be problematic.” “The stigma of cannabis left years ago. Doctors, social workers, other professionals are using and encouraging others to use, which confuses the situation further.”

#### Service providers’ practices

A little more than one quarter (26%) of participants reported a high level of familiarity in screening or assessing cannabis use, while 31% reported a low level of familiarity. Most participants (86%) believe that they have a role and responsibility to screen or assess cannabis use in youth. However, less than half (48%) of participants reported that they often screen or assess cannabis use in youth, 27% do so sometimes, and 23% rarely or never. In terms of treatment of cannabis use, 16% reported a high level of familiarity, 45% moderate, 37% low, and 2% did not respond. Only 16% of participants reported working with families often, 67% sometimes or rarely, and 15% never work with families. When asked about the screening or assessment method, there was a wide range of responses: 40% of participants reported asking structured interview/assessment questions to youth and/or families, 22% wait for the youth or family member to raise the topic before asking about cannabis use, and only 10% use a standardized instrument. Table 3 provides a summary of salient themes linked to screening, assessment, intervention, treatment, and standards of practice, along with frequencies and exemplar quotes.

**Table 3:** Service providers’ practices related to youth cannabis use from survey data

Themes	Percentages	Exemplar quotes
Screening and assessment	26% report high, 43% moderate, and 31% low familiarity with screening 48% report screening/assessing often, 27% sometimes, and 23% rarely or never screen or assess 86% state it is their responsibility to screen or assess cannabis use in youth	“We need more tools on how to screen, and it should be required especially for public health nurses working with infants and young parents who are using.” “We don’t have a standard screening process at work, and need more training on how to respond when youth say that it is used to cope with mental health, and it is legal which they think means it is safe.” “It is hard to talk about this with youth and other professionals because it seems very political and people get very upset when you have a different opinion. It makes me uncomfortable to ‘assess’ this unless I have a really good rapport with the youth.”
Intervention & treatment	16% have a high level of familiarity with treating a cannabis use problem in youth 72% report there are inadequate services and treatments for youth using cannabis	“I don’t know any services that treat cannabis specifically in our town although we are in a rural community with few services and usually refer out of town for inpatient services at a general substance use program.” “No services for cannabis. We are watching kids with mental health problems getting worse and have no services.” “I don’t know how to work with a youth who doesn’t think it is a problem, also what to do when a youth has schizophrenia and keeps using cannabis.” “I don’t treat cannabis and have nowhere to refer my patients once it has been assessed.” “Mothers and fathers who are pregnant using cannabis or just cannabis in the household. How much use by parents is too much, cause for concern, [when to] call CAS?” “Not knowing what to do when they don’t think it is a problem or how to ask questions without judging.”
Working with families	67% rarely or never work with families 15% often work with families	“Our centre offers little for families and doesn’t want us to offer individual sessions. They are referred to a 4-week family group and nothing else.” “I am just not sure how to include families as our services do not really welcome families especially when youth is over 18.”
Approaches, guidelines & standards of practice	18% have high level awareness of professional guidelines or standards of practice related to cannabis	“Not aware of my college or association guidelines and recommendations.” “Need more info on safer use guidelines/harm reduction for cannabis use and youth.” A lack of good macro level supports (schools, hospitals, community level, etc). Macro issues tend to be what cause the problems (poverty, racism, patriarchy, etc. and neoliberalism in general) “I don’t feel like I have enough training or experience in harm reduction.”

#### Service providers’ recommendations

In response to an open-text box question, participants provided recommendations related to youth cannabis use, which were grouped in six categories: (1) increase information on youth cannabis use including risks and harms, (2) enhance training and education of service providers, (3) improve regulation and policies to protect youth, (4) reduce stigma and minimization, (5) use anti-racist, diversity, equity, and inclusion approaches in addressing cannabis use in youth, and (6) increase equitable access to mental health and addiction services for youth and their families. Table 4 summarizes the survey recommendations with exemplar quotes for each recommendation.

**Table 4:** Service providers’ recommendations related to youth cannabis use

Themes	Exemplar quotes
Increase information on youth cannabis use, and its risks and harms	“I don’t think there is a lot of information on risks. There is a lot of information about medical cannabis lately which makes it seem like it is not harmful. I operate from a harm reduction perspective, so I do not advise young adults not to use cannabis.” “Lack of information altogether, in terms of evidence-based studies. It was legalized without enough education in terms of risk. Young parents using while they have kids in their care, without thinking anything of it. Same with using and driving. No idea about the risk of developing psychosis and the long-term consequence of that.” “[More] on adverse effects. [More] statistics on onset of psychotic disorders caused by cannabis. [Also] the number of young people ending up in ER due to cannabis induced symptoms (i.e., hyperemesis, respiratory problems, psychosis).”
Enhance training and education of service providers	“Need to provide training and education for front line staff who work with youth to ensure that use of cannabis is part of the assessments and treatment plans.” “Professional development for school mental health providers (psychology and social work) regarding harm reduction, stats about current use/abuse and how legislation has increased access to cannabis (store at every corner it seems).” “The support/education is left up to mental health providers (and medical) now that it has been legalized rather than the government taking on that responsibility. Having any structured education/ resources would be extremely beneficial.” “Many of my clients (who are primarily queer and trans) use cannabis and they tend to consider it self-medicating. Many of them have come to use cannabis to regulate sleep and anxiety…I want to respect that but a part of me doesn’t fully understand the physiological and psychological impacts of cannabis use on the brain for youth…It’s difficult to know how to fully educate my youth clients when I’m not 100% sure where I stand on the issue. More information would be helpful to fully inform my clients.”
Improve regulation and policies to protect youth	“Raising the legal age would be helpful.” “More regulation, limit on number of dispensaries, and improve policies around how cannabis is promoted.”
Reduce stigma and minimization	“Substance use is poorly integrated into mental health services and they often perpetuate stigma, judgment, and shame in their efforts to support people seeking services.” “It is a one-sided conversation because we are viewed as not progressive enough and stigmatizing if we disagree with the way cannabis use is normalized and even glorified.”
Use anti-racist, diversity, equity, and inclusion approaches in addressing cannabis use in youth	“Anti-Black racism and colonialism need to be understood when intervening around cannabis because those systems shaped present day and historical legal and policy approaches to it.” “I think cannabis is not stigmatized and actually normalized, but for some youth like the Indigenous youth in our community, there are a lot of assumptions and stigma when they are getting services in non-Indigenous treatment centres.”
Increase equitable access to mental health and addiction services for youth and their families	“Most youth don’t have access to therapy at all and if they do, it’s typically time-limited and often not culturally relevant or trauma-informed.” “We need more services for cannabis use. Youth are dealing with so many difficulties and while it is clear that marijuana is causing problems, they are sometimes dealing with poverty, homelessness, and more fatal substance use like opioids so it sometimes seems to be less critical than the other issues.”

### Focus group results

A total of 12 service providers participated in one of two focus groups, with four participants in one focus group and eight in the other. Most focus group participants were social workers (n = 11), and one participant was a child and youth worker. In terms of years of experience, 33% of service providers had less than five years of experience working in the field of mental health and addictions, 25% with 6–10 years of experience, 25% had 11–20 years of experience and 17% had more than 21 years of experience. A high percentage of participants identified as women (75%), 17% identified as gender diverse including nonbinary, trans, gender fluid or gender queer, and 8% identified as men. Participants ranged in age, with 8% between 20–29 years old, 58% between 30–39 years old, 17% between 40–49 years old, and 17% between 50–59 years old. In terms of racial identity, most participants identified as white (83%), followed by Black (8%), and mixed or biracial (8%). Participants spanned four geographical regions of Ontario, with 58% in Central Ontario, 17% from Southwestern Ontario, 8% from Eastern Ontario, and 8% in both Northwestern Ontario and Central Ontario. In terms of work setting, 75% of participants were in the public sector and 25% were in the private sector. Half of the focus group participants (50%) worked in community agencies, 17% worked in hospitals, 17% worked in private practice, 8% were in both hospital and private practice, and 8% were in other settings (e.g., high school). Table 5 provides a summary of characteristics of participants in the focus groups.

**Table 5:** Focus group participant characteristics (*N* = *12*)

Characteristics	Focus group participants n (%)
Profession	
Social worker	11 (92%)
Child and youth worker	1 (8%)
Experience working in health, mental health and/or addictions	
5 years or less	4 (33%)
6–10 years	3 (25%)
11–20 years	3 (25%)
21 + years	2 (17%)
Gender	
Woman	9 (75%)
Man	1 (8%)
Gender diverse (queer, gender fluid/queer, male queer, female queer, non-binary, gender independent, gender diverse, trans man/trans masculine/man of trans experience)	2 (17%)
Age	
20–29 years old	1 (8%)
30–39 years old	7 (58%)
40–49 years old	2 (17%)
50–59 years old	2 (17%)
Racial Identity	
Black (African, Afro-Caribbean, African Canadian)	1 (8)
White	10 (83%)
Mixed race	1 (8%)
Religion	
No religion	5 (42%)
Christian	4 (33%)
Catholic	1 (8%)
Atheist	1 (8%)
Wiccan	1 (8%)
Region	
Central Ontario	7 (58%)
Eastern Ontario	1 (8%)
Northwestern Ontario	1 (8%)
Southwestern Ontario	2 (17%)
Multiple regions selected (Northwestern Ontario, Central Ontario)	1 (8%)

*Note*. Percentages may not total 100 due to rounding. Focus group participant characteristics were self-reported

**Fig. 1:**
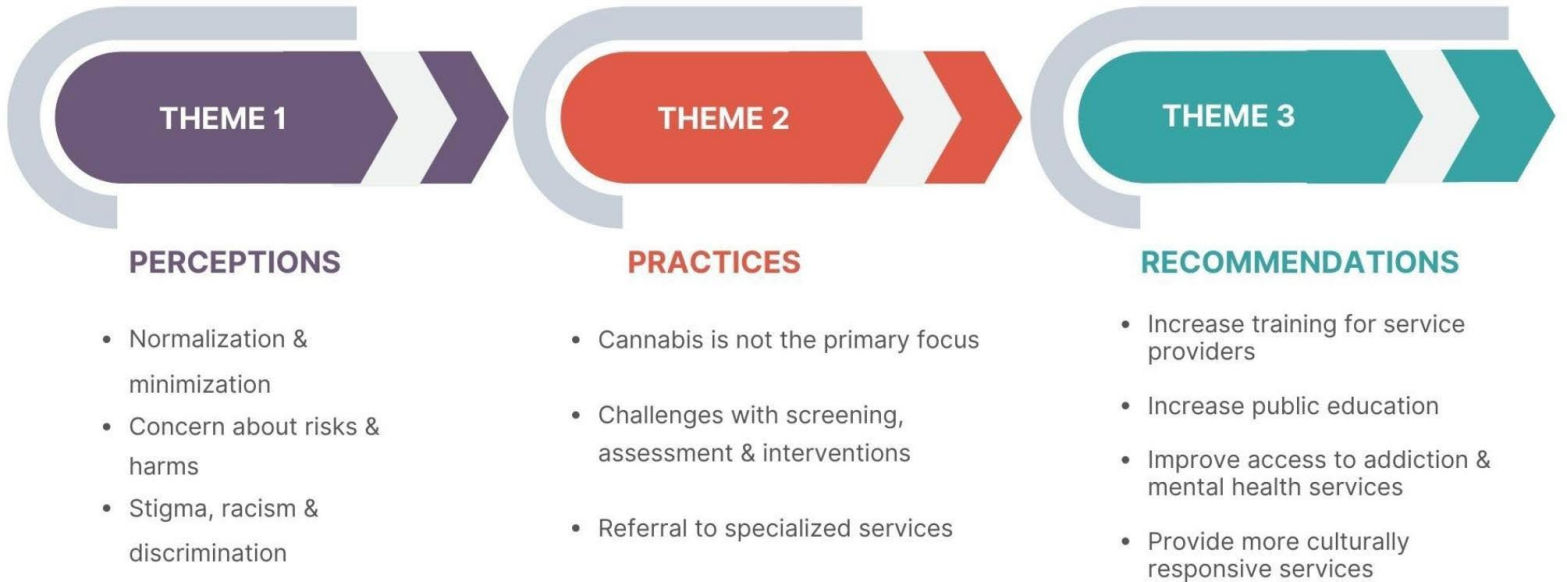
Focus group themes and subthemes

The focus group data consisted of three themes that were established a priori: *perceptions*, *practices*, and *recommendations*. These themes consisted of several subthemes that emerged during the analysis (Fig. 1).

Under the theme of *perceptions*, the first subtheme was normalization and minimization of youth cannabis use. Service providers noted that many youth and other service providers tend to assume that natural means safe. They described how cannabis use among youth is often normalized, using terms such as “normal”, “natural”, and “less harmful” when discussing cannabis or its use in comparison to other substances. They also highlighted the challenges they face in addressing youth’s perceptions of cannabis as a natural substance. As one service provider stated, “It’s perceived to be a natural substance, you know, from the earth, it’s not chemically based. It’s perceived as less harmful than other ways of maybe achieving similar effects from other substances” (P2).

Service providers also noted that some youth believe that cannabis is more helpful than medication. One participant mentioned, “A lot of people feel way more comfortable smoking and using marijuana than they would taking anti-anxiety or anti-depressants, feel that it’s more natural and that it helps them more” (P12). Additionally, some service providers normalized cannabis use as a typical part of development for youth and drew comparisons to their own lived experiences:

This is an age, a time of a young person’s life where they’re trying things. I came home with piercings; I came home with different hair colours…we have to remember when we were young…the wild things that we all did, and we all came out okay. (P5)

Another service provider highlighted biases among social workers who minimize youth cannabis use based on their personal experiences. This service provider explained, “There’s kind of a conflation of, like, oh, well, I use [cannabis] and there’s nothing wrong with it, so it’s fine for my teenage clients who use” (P3).

Some service providers expressed that, in comparison to other substances, cannabis is perceived as less harmful. One service provider provided an explanation:

A lot of the clients that we see are quite heavy into other substances that tend to have more immediate and negative side effects, like alcohol and harder drugs, and some youth will smoke cannabis instead of drinking. And from a harm reduction point of view for us, like we know that long term, that can be really detrimental for their brain development, amongst other things, but in the here-and-now, a lot of them, if they go out and get drunk, they can’t come to school, or they end up interacting with police, or they end up in the hospital, or they end up attempting suicide. Whereas if they smoke up, they come to school and they just hang out with their friends…from that point of view, it’s not great, but it’s less harmful than some of the other things they could be doing. (P11)

The second subtheme under the perceptions theme is concerns about risks and harms. Most service providers expressed concern about the adverse effects of cannabis use, particularly in relation to brain development. One participant stated, “Youth ages 16 to 24, that’s such a crucial time pf their lives when their brains are still really forming” (P6). Service providers also worried about increased anxiety, psychotic episodes, exacerbation of eating disorders, and risk of addiction or misuse of other substances. Another participant emphasized, “the risk of developing serious mental health concerns related to cannabis use is much, much higher before the age of 25” (P9). Additionally, service providers expressed concerns about cannabis use preventing youth from learning how to regulate and cope with intense emotions, as well as its impact on motivation and “falling behind in school” (P3). One participant mentioned that cannabis use can also increase risk-taking behaviours and create conflict in family relationships, “I've seen it a lot in the parent-teen dynamic, where it’s created quite a lot of strain in the relationship” (P3).

The final subtheme under the *perceptions* theme is stigma, racism, and discrimination. Service providers described mixed perspectives related to stigmatization of cannabis use. While some believed that the “stigma around cannabis use has declined a lot,” (P9) many acknowledged that youth experience stigma differently depending on their intersectional identities. One service provider shared their perception:

I don’t actually see [stigma] impacting them that much, and I think that has more to do with the fact that they have so many other aspects of their lives, like who they are, that are impacted by stigma, that cannabis is kind of, like, the least of their stigma concerns. So, the stigma that impacts them is more around race or more around their age, or more around the other substances that they might be using, and not whether or not they’re using cannabis. (P11)

Another service provider discussed how youth of colour are coping with structural and systemic racism, leading to inequitable treatment across various services and systems:

I have seen in my practice that when other young people use cannabis, there’s a question of what is going on in their life and they’re met with compassion, they’re met with, okay, connecting them with a counsellor and mental health supports, and then I’ve seen racialized children streamlined into the criminal justice system. They are not given extrajudicial measures, meaning that they are not met with a practice of, okay, how about we not give them an arrest that can lead to a criminal record. (P5)

Another service provider highlighted stigma occurring at the intersection of ethnicity, gender, and substance use:

So, somebody that smokes cannabis on a regular basis, you know, if they’re a person of colour, or if they’re a female, they may get treated more - what’s the word? They may be treated differently, but they may say, well, that’s not how a girl should behave or it’s worse for a female, say, to look high or to behave or to act like they’re high, than it is for a man, and that increases, let’s say, the perception of that injustice or that kind of differential treatment between males and female. (P7)

Stigma related to cannabis use and socioeconomic status was identified, although service providers acknowledged that cannabis use “does not seem to be in any one place. It’s kind of kids from all economic areas” (P10). However, they noted that youth with lower income “do not necessarily have [treatment] resources available to them” (P2) and “may be more highly stigmatized because of their income” (P3). One service provider also described stigma towards youth with learning disabilities who use cannabis: “I’ve also seen stigma a lot with clients who have learning needs, so like learning disabilities oryouth who are on education assistance plans in the school…they’re treated differently” (P8). Another service provider stated, “practitioners just lose the strengths-based approach when we find out that young people use cannabis” (P1).

Another service provider spoke about stigma-related “challenges in a big institution…in particular, where the medical model is the dominant mode of practice” (P4). They described a youth’s experience in a hospital emergency room:

[Youth] was sitting on a bed, waiting to speak to a doctor, and there were two mental health crisis nurses chatting together, just outside [youth’s] closed curtain of [youth’s] bed…a really derogative statement was made…‘Those are a waste of humanity’… as if their humanity has less value or less worth…There is a lack of education, a lack of sensitivity and empathy, and mostly fear. Fear is present because of the lack of education and training…And so, this kind of idea that we can be quick to dispose of those people or get them out of our sight, because they’re scary and they’re dangerous. So, to me, this to me is like a failure of our society. (P4)

The theme of *practices* had three subthemes, and the first described how cannabis is not a primary concern for youth, service providers, and outpatient services. Most service providers noted that cannabis “doesn’t tend to be the presenting issue” in their mental health practice when there are “complex mental health, anxiety, depression…ADHD…and stopping using cannabis is kinda the last of people’s concerns” (P12). According to one service provider, “the kids that I see have to identify what goals they want to work on. So, unless somebody specifically tells me, at some point, that they want to change something about their cannabis use, it’s not necessarily the focus” (P6). Another service provider stated, “It is not the focus of the counselling in the mental health outpatient department of the hospital…it kind of comes up as almost like a sidebar. I'm aware that I’m not asking them to expand a lot on that” (P4).

The second subtheme under *practices* involved challenges with screening, assessment, and interventions. Most service providers described not screening for cannabis use and not including cannabis use (or other forms of substance use) as a standard part of their assessment. They indicated that they expect youth to raise this if it is a presenting concern, as noted by this service provider: “Young people are very honest. So, I’ve never had to assess, they just outright have said it” (P5). One service provider stated that they do not offer counseling that addresses cannabis use: “The talk that we have about cannabis is a side thing. I don’t offer direct counselling or support” (P7). While Motivational Interviewing (MI) and harm reduction were identified as guiding models or approaches used by service providers, these appeared to be applied sporadically. For example, one service provider stated that they provide “Motivational Interviewing in general, to kind of see what [youth] thoughts are on the situation and kind of, you know, look at the good, the bad, and all the rest” (P2). This service provider also explained that “you can’t be abstinence-based when it comes to offering youth work” and that this is “denying them service” and the “only way you’re gonna engage them in service” (P6). However, there was a lack of clarity on how harm reduction guidelines are incorporated in practice. One service provider stated, “I'll do a quick, kind of, safety spiel to make sure that they know what some of the harms are” (P10). Another added, “what I generally do is kind of try and just provide a bit of psychoeducation in terms of harm reduction” (P12). A few service providers described the importance of psychoeducation and “supporting the youth in the choices that they want to make around their cannabis use” (P11). Another service provider explained that it is important to “take a look at [youth’s] cannabis use, and how it might have impacted them coming to hospital and just providing a little bit of education” (P2). Some service providers underlined not having the necessary knowledge and skills to work with neurodiverse youth using cannabis: “I'm currently finding it a struggle to support tapering or support, like, even working towards abstaining with cannabis use with clients with autism” (P8).

The final subtheme under *practices* focused on referral to specialized services. Many service providers indicated that they refer youth who would like to address their cannabis use to specialized services due to low confidence in treating this directly. One service provider stated, “If we recognize that it’s a situation that the client wants to get out of, and it’s become abuse, not just use, recreational, my confidence is so low, I'd refer them out, simply” (P7). Another shared, “I actually don’t know that much and so, I do have a tendency to refer people out. But you know, I think it would be really good to feel a bit more competent” (P1). This lack of confidence was described by another service provider:

There is still the idea that it’s someone else over there who can handle it better, or who really has the real skills…if we are seeing people [who] are identifying cannabis or other drug use as a problem, we might make a referral to [name of local agency], which is like our local agency that does addiction-specific counselling, instead of, perhaps, feeling confident to educate the individual about what we know about cannabis use and risk. (P4)

The third theme was *recommendations*, and it included four subthemes, with the first focusing on the need to increase training for service providers. There was a unanimous call for increased training and education on youth cannabis use by all service providers: “I think the problem with cannabis, is that everything is very vague…for me, the biggest barrier in feeling competent in addressing it, is knowing the specifics, or even the specifics being known, in general, from larger scientific community” (P7). Most service providers expressed a desire to learn more and underlined that this would improve their practice, as stated by this service provider: “I’d be very interested in learning a little bit more on the neurological impacts of cannabis use, on developing brains, but also on why there is that connection between cannabis and psychosis or paranoia. Sometimes if you can explain the ‘why’, it’s more effective” (P6). Most service providers indicated that increased training would enable them to provide more psychoeducation to youth who report that cannabis is beneficial. As noted by one service provider:

I sometimes get a little confused when, you know, young people are talking about using cannabis to deal with anxiety…a 17-year-old walks in and says, ‘yeah, I got anxiety, I just get high’. Like, they don’t know - I don’t know if that’s helpful - I guess it could be, if you happen to get lucky and you have a strain that works for you. (P10)

Some participants expressed confusion about the different cannabis products on the market and not knowing the various effects of each one: “They can help to do this or do that, but can they harm? In terms of understanding what are the different strains, what are the different intensities, what cannabinoid is responsible for what?” (P7) Some service providers were unaware of existing low-risk guidelines for cannabis and underlined the need for “more education on how to safely use cannabis and who is supposed to be using cannabis” (P5). Many of the service providers compared cannabis with alcohol in terms of safety guidelines, explaining that they would like to see more tools to educate youth about safe use:

We’ve been able to study [alcohol] and gain all this knowledge in terms of, like, withdrawal skills and how do you kind of predict different behaviours based on intoxication levels and you can use that as a tool to kind of teach your patients around, like, moderation and kind of education in that way. There’s not really an equivalent that I’m aware of for cannabis. (P2)

The second subtheme under *recommendations* highlighted the critical need to increase public education on cannabis use and its effects on youth. Most service providers expressed frustration about the “lack of education around legalization and particularly, the lack of discussion and education with this age group” (P4). As explained by one participant, “I don’t think, again, this age group is being educated about the actual impact on their development. I'm not sure parents know either. As a parent of a teen, I don’t think I know everything I should” (P4). Some service providers recommended that schools are an important place where information can be given at a younger age. For example, “we have an initiative with our school boards to provide substance use education as a part of their health classes. But we’re now seeing that maybe that needs to go down to lower grades, to provide education sooner” (P8).

The need to improve access to addiction and mental health services was a third subtheme underlined by most service providers. Some service providers described not being able to refer youth to services within their own communities, particularly in remote or rural areas of Ontario. “We’re having to send youth really far places to get day treatment, at hospitals that provide it, outside of our region…I'm thinking more funding and policies around, like, interventions in more community settings, I think, would be really helpful” (P8). According to one service provider, “we have, like, wait times for mental health supports going into one year, one year and a half. And so, what are they supposed to do in between that time?” (P5).

The final subtheme focused on the need for more culturally responsive services that are equitable and apply anti-oppressive approaches. Service providers explained that “connecting [youth] to culturally relevant services” (P6) which recognize intersectional identities, ethnoracial-religious values, as well as the experiences of systemic racism and discrimination, creates safer spaces to discuss cannabis use. They discussed the need for anti-racist and culturally responsive services not only in healthcare but also in other sectors such as school and legal systems. One service provider explained, “racialized young people, Black and Indigenous, specifically, I have found that they are not treated with the same compassion as their counterparts by the school system and by the criminal justice system” (P5). Service providers discussed hearing about inappropriate comments made by hospital staff when a youth using substances (including cannabis) presents at the emergency, and they recommended having “[cultural] sensitivity training with emerg staff, particularly” (P4). Service providers also underlined the importance of ensuring that *all* aspects of service delivery are inclusive and equitable. One service provider described the significantly negative impact of agencies or hospitals requiring youth to complete forms that are not using gender-inclusive language: “The intake form only had M or F written down…I’ve had young people tell me ‘I'd rather die before going to these places for help’” (P3).

## Discussion

This study makes an important contribution to the sparse literature on service provider perceptions of youth cannabis use in Canada and provides insights about their practice, as well as recommendations to improve policies, practice, and education. Our findings drew from quantitative and qualitative survey data, as well as qualitative focus group data. A mixed method design was an appropriate method that illuminated complementary findings across the quantitative and qualitative data. Data from both the survey and the focus groups showed gaps in service provider knowledge and skills that impact many aspects related to practice, including assessment and intervention. Some of the responses from service providers showed uncertainty or inconsistency between perceptions and practices. Many service providers reported that cannabis use can negatively impact youth mental health, but they also underlined that it is difficult to address this when many of the youth they serve are using cannabis to cope with mental health symptoms. Most service providers believe that they have a role to screen or assess cannabis use among youth clients, but less than half screen or assess regularly in their practice. Furthermore, few service providers use standardized instruments, many wait for youth to initiate the discussion on cannabis use, many do not believe that cannabis use is a primary focus of treatment, and many refer youth with cannabis use concerns to specialized services. Similarly, most service providers recognize the important role of families in youth treatment, but most do not work with families.

These results highlight gaps in knowledge, skills, and confidence of service providers related to youth cannabis use, and substance use studies have shown that one of the primary reasons for these gaps is inadequate training and education [43, 61, 62]. Turuba et al. (2022) [63] explored the perceptions and experiences of youth aged 12–24 engaged with substance use services in British Columbia, Canada. Youth described needing more information about the risks and potential harms associated with substance use, and they reported that service providers disregarded their cannabis use and did not recognize that it can be an addictive substance for some youth. The study underlined the importance of screening and brief interventions because youth may not mention their cannabis use unless they are asked directly by the service provider. Substance use research has also shown that it is important to adopt an integrated approach to address co-occurring mental health and substance use concerns, which can facilitate the identification of cannabis use and other substances in youth [63–65].

Most service providers reported not having a strong understanding of cannabis for medical purposes. Some referred to cannabis as natural and less harmful than other substances, while other service providers found it challenging to respond to statements made by youth that it is natural and medicinal. Since 2001, Canadians have had access to cannabis for medical purposes [66]. In 2016, the regulatory framework was reviewed, and the government introduced the Access to Cannabis for Medical Purposes Regulations (ACMPR), which provided increased access to cannabis with the authorization of a health care provider [67]. More than 30% of people report using cannabis for medical purposes [68], but almost 75% do not actually have medical authorization from a health care provider [69]. According to the CCSA, “the current evidence does not yet suggest that cannabis and cannabinoid products are effective for treating many of the health conditions for which claims are being made” [42 p6] which has also been cited by other sources [70–73]. The Canadian Pediatric Society cautions that the therapeutic use of cannabis can also carry significant adverse effects; therefore, this should be evaluated on a case-by-case basis with careful consideration and discussion of risks and benefits [74]. It is important that service providers have more accurate information about cannabis for therapeutic purposes.

It is important to note that 69% of the service providers in this study were social workers, which is not surprising when we consider that this profession is one of the most prevalent regulated professional groups in Ontario that focuses on mental health. In Ontario, there are approximately 23,757 social workers [75], 9,220 Registered Psychotherapists [76], 4,373 psychologists [77], 3,820 social service workers [75], and 1,938 psychiatrists [78]. Similar to other professional programs, there is a need to increase the training on addictions in social work programs to better meet the needs of individuals and families seeking mental health and addiction services [79–81]. One study found that there are no required courses on addictions in any of the Canadian schools of social work and only one-third of social work programs offer elective courses [79].

In addition to the need for increased service provider training, our study showed the urgent need for public education on cannabis use, which is a clear message by the CCSA highlighting the importance of “education and awareness” for “informed choices”.[42 p5] The gaps in public education, despite the high rates of cannabis use in youth, may explain some service providers’ disagreement or ambivalence about legalization. Many service providers underlined that legalization of recreational cannabis came into effect with inadequate public education, inadequate training for service providers, and inadequate access to addiction services to protect children, youth, and young adults. Most service providers were unfamiliar with lower-risk cannabis use guidelines and that there are lower risk guidelines that have been developed for youth [82]. While many spoke about their support for harm reduction approaches, more than half of service providers do not screen for cannabis use, only a small percentage of service providers are familiar with how to treat cannabis use concerns in youth, and many refer youth reporting concerns about cannabis use to specialized services, which indicates that potential harms from cannabis use are not adequately addressed and reduced. Gunderson et al. [61] had similar findings in an American study exploring school service providers’ experiences working with youth with cannabis use concerns. The study called for increased training on how to counsel youth with substance use concerns (including cannabis) and recommended the development of a youth-focused harm reduction protocol that may strengthen service providers’ interventions.

Another important finding from our study relates to stigma and normalization. Less than half of the service providers believe that youth using cannabis are stigmatized, but 38% expressed that there is no stigma and attribute this to the normalization of youth cannabis use. They also noted that the normalization of youth cannabis use has made it difficult to express concerns about the adverse effects of cannabis use for this age group. Stigma is defined as negative stereotypes, assumptions, or attitudes toward individuals or groups [83]. Legalization has been associated with increased social acceptability [2, 84], and reduced perception of risks and harms [19, 39]. Health knowledge and perceived risks influence the rates of cannabis consumption, with higher rates when a substance is not perceived as harmful [39, 85]. According to Ali et al. [84] stigmatization and normalization are two dominant discourses associated with youth cannabis use in Canada, and this carries important public health implications. The normalization theoretical framework conceptualizes a process whereby drug use becomes less stigmatized, and there are shifts in cultural attitudes and behaviours related to youth drug use, prevalence rates, access, and availability of drugs, as well as policy changes [86, 87]. Asbridge et al. [88] compared the normalization of cannabis with the denormalization of tobacco. Denormalization involves an adoption of new values that no longer support or perceive the behaviour as legitimate or mainstream. Policy and education played a key role in the denormalization of tobacco (i.e., access, availability, marketing, packaging, stigmatization, age restrictions, restrictions on where people can smoke, and sales restrictions), which shifted social and cultural norms. The authors found that perceptions of health risk shape people’s experiences of normalization and denormalization. This study did not focus on youth specifically and was pre-legalization in Canada, but the findings showed that participants had more concern about the serious health risks associated with tobacco use while cannabis use was viewed as less harmful, presumably given little public education about health risks associated with cannabis use. Ali et al. [84] explained that normalization of cannabis use without adequate education can minimize the perceived harms linked with cannabis use and contribute to increased use. The normalization framework does not consider inequities, racism, and discrimination, and in response to this important shortcoming, researchers introduced the term “differentiated normalization,” which recognizes how social and structural locations influence the extent to which cannabis use is normalized, stigmatized, and criminalized among different groups [87, 89, 90].

There was consensus among service providers in this study that youth from equity deserving groups are more stigmatized when they use cannabis and other substances. A conceptual paper on substance use stigma underlines the importance of an intersectional analysis that focuses on “social identities, systems and structures that interact to produce privilege and oppression”.[83 p89] A review by Public Health Ontario (2022) [91] found that there are many factors underlying race-based health inequities in substance use including stigma, complex pathways to care, mistrust of the healthcare system due to systemic racism and discrimination, and the absence of culturally informed services. There has been a long history of racial disparities in cannabis arrests for Black and Indigenous people in Canada pre-legalization [92], and one of the other purposes of the Cannabis Act is to reduce cannabis-related criminalization among youth [3]. Callaghan et al. (2021) [93] found that there have been short-term reductions in cannabis-related criminalization of youth (12–17) with the new legalization framework that allows youth to possess small amounts of cannabis. However, the study does not provide disaggregated data by race and ethnicity, warranting the need for further research to know whether there have been reductions in cannabis-related criminalization for youth from equity deserving groups.

Service providers in our study described the need to reduce barriers to mental health and addiction services and increase equitable access to quality care for youth and their families. Access to mental health and addiction services is a longstanding issue that has been amplified in Ontario during the pandemic due to lockdown measures that closed or restricted the delivery of services [94, 95]. A recent study found that structural barriers such as approachability, availability, affordability, appropriateness, and acceptability, impact access to mental health and addiction services for Ontario youth [95]. Service providers in this study described the importance of *acceptability* of services, which refers to the extent to which services are culturally responsive and meet the diverse needs of youth and families. Culturally responsive services require organizational practices, treatment modifications, and treatment access that focus on (1) cultural values in a setting and treatment, (2) demonstration of cultural humility, as well as anti-racism and anti-oppressive interventions, (3) culturally adapted evidence-informed interventions, and (4) innovative strategies aligned with cultural values [96]. Studies have shown that individuals with unmet healthcare needs often report acceptability of services as the most common structural barrier [97, 98]. Integrated youth services have been proposed and implemented as a response to these service gaps and system challenges globally [99, 100]. In Ontario, there is a growing movement to address system shortcomings through co-designing services with youth to enhance their acceptability to youth through Youth Wellness Hubs Ontario [101].

## Limitations and strengths

This study has some limitations including the fact that its findings may not be generalizable across the country as the study was conducted in Ontario and policies, services, and education vary across the provinces. While the study included eight different professions, there was an overrepresentation of social workers, and the findings may not be generalizable to other mental health professions. One of the strengths of the study was the used of mixed methods, which provided breadth and depth on service providers’ perspectives. Another strength was the diversity of service providers in terms of profession, years of experience, gender, age, race, religion, and region of Ontario.

## Conclusions

This study highlights service providers’ perceptions, practices, and recommendations related to youth cannabis use with important policy, education, and service implications. Youth cannabis use in Canada is a public health concern that needs to be addressed through increased public education on risks and harms. Service providers need increased education and training to enhance their knowledge, skills, and confidence in addressing cannabis use. There is a pressing need for expanded education for youth and parents, improved access to mental health and addiction services, and the development of more family-centred and culturally responsive services. The legalization of cannabis was intended to protect youth and reduce their access to it. However, this objective has not been fully achieved, emphasizing the critical need for a public health approach to reduce prevalence rates of cannabis use among youth and mitigate associated harms.

## Data Availability

Not applicable.
